# Obesity care: more urgent than ever

**DOI:** 10.20945/2359-3997000000327

**Published:** 2021-02-24

**Affiliations:** 1 Universidade Federal de Santa Catarina Florianópolis SC Brasil Universidade Federal de Santa Catarina, Florianópolis, SC, Brasil.

Over the past few decades, the world population has faced an obesity pandemic, closely followed by a progressive increase in the prevalence of other chronic non-communicable diseases. The Global Burden of Disease study estimated that obesity was responsible for about 4.7 million premature deaths worldwide in 2017 (1). In 2020, another pandemic of catastrophic proportions overlapped this scenario: COVID-19, an infectious disease caused by severe acute respiratory syndrome coronavirus 2 (SARS-CoV-2). At the time this editorial was written, nearly 2.4 million deaths by COVID-19 had been recorded worldwide, of which more than 238 thousand were in Brazil (2). This coincidence of two pandemics tends to be especially harmful in regions where economic and social inequalities predominate, since consequences of both COVID-19 and obesity are disproportionately worse in marginalized groups (3).

Obesity, as well as its comorbidities, is a known risk factor for the severity of COVID-19, including increased risk of hospitalization, need for intensive care and death (3). In this issue of *Archives of Endocrinology and Metabolism*, Brandão and cols. (4) review the pathophysiological mechanisms that interconnect obesity and COVID-19, with a focus on inflammation and abnormal production of cytokines, a phenomenon known to be associated with both conditions. They explore how SARS-CoV-2 infects cells and exerts its pathogenic effects, in addition to changes in the host's immune system that predispose to the so-called “cytokine storm”. Obesity, especially when there is ectopic fat deposition, is associated to immune system dysfunction and predisposes to other conditions such as diabetes, respiratory, cardiovascular, liver and kidney disorders that also result in an increased risk for severe forms of COVID-19.

The authors finally list preventive and therapeutic approaches that could minimize the impact of the meeting of these two pandemics. Among the suggested measures are interventions in eating habits and physical exercises capable of inducing weight loss, albeit modest. Although the impact of weight loss interventions on the course of infections has not been specifically studied, there is evidence that they can reduce inflammation and lead to several health benefits (3,5).

In another review article from this issue, Halpern and Mendes (6) bring an interesting discussion about a popularly used method for weight loss purposes: intermittent fasting (IF). They critically analyze the evidence available to date and show that, like other diets, IF has advantages and disadvantages. The effect on weight loss appears to be directly related to calorie restriction and not to other alleged metabolic effects. Nevertheless, it can be a reasonable strategy for certain patients who would rather follow one of the IF protocols than count calories on a daily basis.

In fact, a number of dietary patterns may be acceptable in order to induce a negative calorie balance and, consequently, weight loss. On average, there is no clear superiority of one method over another, although clinical trials show a high degree of individual variability within each diet group (7). Personal, social and cultural differences, as well as nutritional adequacy must be considered in clinical practice. Therefore, individualized planning by a qualified team is essential.

The challenges of achieving adequate obesity management are even greater nowadays. Social isolation inevitably reduced physical activity levels, and many people have changed their eating and sleeping habits towards a pattern that favors weight gain (8). Furthermore, the COVID-19 pandemic imposed a reorganization of health services, which negatively impacted health care of patients with chronic diseases (9).

Public health policies involving educational, regulatory and tax measures are essential in preventing excessive weight gain, however the results of this type of intervention are not expected in the short term. In parallel, the implementation of measures that strengthen the health system, especially in primary care, are important to reduce the burden of the disease. Continuous interaction with health care providers is one of the main determinants of long-term success when treating obesity (7). People with obesity must have access to trained multidisciplinary teams who provide them with evidence-based treatments and follow-up (3). Given the physical distance recommendations after the beginning of the COVID-19 pandemic, especially for groups at higher risk, telemedicine can be a useful tool to replace some of the in‐person office visits. Although there are no long-term studies, two clinical trials using telemedicine-based obesity counseling have been shown to improve adherence and weight loss in 12 weeks (10,11). It is important to emphasize that the focus should be on improving care, and not on weight loss campaigns that warn of obesity risk and hold the individual responsible, since they have proven to be ineffective and even detrimental (12).

There is definitely a lot to be done. The spread of SARS-CoV-2 infection further exposed the fragility of many health systems in relation to care for chronic non-communicable diseases, especially obesity. In order to reduce the extensive damage that the COVID-19 pandemic has been causing, comprehensive and qualified obesity care is now more urgent than ever.
